# Supplementary material for: Computer-assisted analysis of polysomnographic recordings improves inter-scorer associated agreement and scoring times
Source: PLoS One. 2022 Sep 29;17(9):e0275530. doi: 10.1371/journal.pone.0275530 (PMC9522290; doi:10.1371/journal.pone.0275530)
Supplement: S1 Appendix — (DOCX) [file pone.0275530.s001.docx]

**S1 Appendix. Scoring time individualized per-recording and per-scorer analyses**

Supporting information regarding main manuscript:

“**Computer-assisted analysis of polysomnographic recordings improves inter-scorer associated agreement and scoring times”**

Diego Alvarez-Estevez, Roselyne M. Rijsman

Common notation considerations: data distributions are characterized using the five-number summary as *p50 [minimum, p25, p75, maximum]*, where *XX* in the *pXX* notation refers to the corresponding percentile value. For further details, see Methods section in the main manuscript.

Table A1. Comparison of scoring time differences for sleep staging between manual and semi-automatic scoring approaches at the recording level

| **Sleep stating** | **n** | **Manual** | **Semi-Auto** | **Gain factor** | **Wilcoxon test *p*-value  (paired)** | **Effect size** |
| --- | --- | --- | --- | --- | --- | --- |
| SN1 | 12 | 40.59 [31.07, 34.72, 58.41, 142.63] | 29.09 [1.33, 19.32, 41.90, 147.76] | 1.40 | 0.0522 | 0.6021 |
| SN2 | 12 | 26.70 [14.91, 20.22, 39.03, 72.84] | 19.49 [5.14, 16.25, 27.93, 96.83] | 1.37 | 0.1294 | 0.3165 |
| SN3 | 12 | 57.14 [32.61, 45.02, 69.11, 166.32] | 48.05 [21.01, 32.51, 64.36, 180.04] | 1.19 | 0.2661 | 0.3616 |
| SN4 | 12 | 25.26 [18.50, 19.44, 34.39, 87.92] | 24.54 [14.31, 22.65, 31.01, 75.56] | 1.03 | 0.5693 | 0.2420 |
| SN5 | 12 | 16.97 [9.12, 13.83, 29.92, 47.88] | 12.86 [9.02, 11.42, 18.08, 36.70] | 1.32 | 0.0210* | 0.8328 |
| Overall | 60 | 32.62 [9.12, 21.74, 48.64, 166.32] | 24.54 [1.33, 16.25, 39.81, 180.04] | 1.33 | 0.0005* | 0.4297 |

**Statistically significant result*

Figure A1. Comparison of individual scorer times for sleep staging between manual and semi-automatic scoring approaches

~~
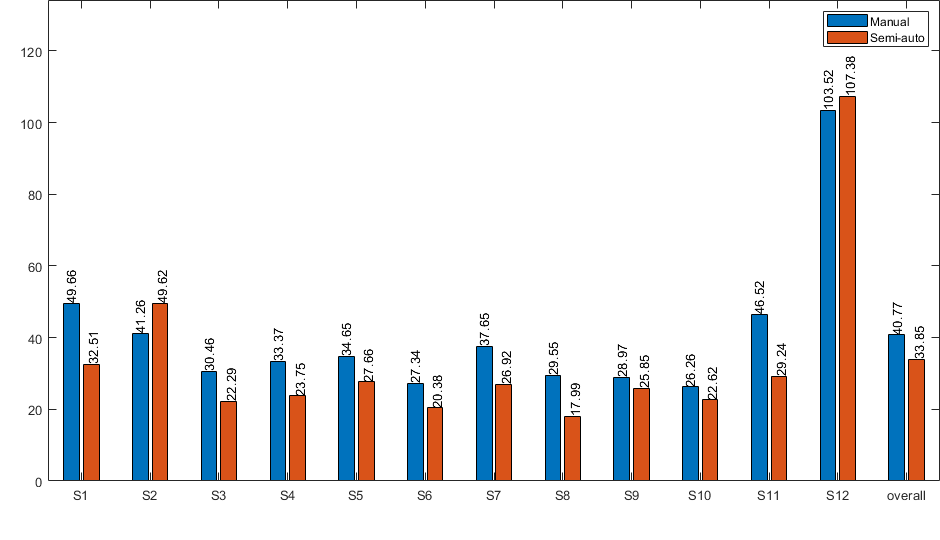
~~

Table A2. Comparison of scoring time differences for detection of leg movements between manual and semi-automatic scoring approaches at the recording level

| **Limb movements** | **n** | **Manual** | **Semi-Auto** | **Gain factor** | **Wilcoxon test *p*-value  (paired)** | **Effect size** |
| --- | --- | --- | --- | --- | --- | --- |
| SN1 | 12 | 47.42 [25.22, 36.11, 65.35, 96.60] | 25.30 [12.16, 18.15, 28.31, 41.56] | 1.87 | 0.0005* | 1.5405 |
| SN2 | 12 | 27.13 [18.37, 20.16, 31.83, 40.69] | 11.40 [8.38, 9.00, 17.64, 31.55] | 2.38 | 0.0005* | 2.0332 |
| SN3 | 12 | 37.03 [23.76, 28.49, 45.91, 71.19] | 14.78 [9.74, 12.03, 17.30, 25.19] | 2.51 | 0.0005* | 2.0719 |
| SN4 | 12 | 68.79 [40.07, 60.49, 84.20, 126.08] | 20.36 [10.61, 15.54, 31.61, 55.67] | 3.38 | 0.0005* | 2.9294 |
| SN5 | 12 | 51.32 [28.04, 39.98, 65.97, 90.48] | 26.43 [12.37, 17.60, 38.51, 109.61] | 1.94 | 0.0210* | 0.9291 |
| Overall | 60 | 44.53 [18.37, 31.00, 65.30, 126.08] | 18.50 [8.38, 12.63, 26.73, 109.61] | 2.41 | < 0.0001* | 1.3475 |

**Statistically significant result*

Figure A2. Comparison of individual scorer times for detection of leg movements between manual and semi-automatic scoring approaches


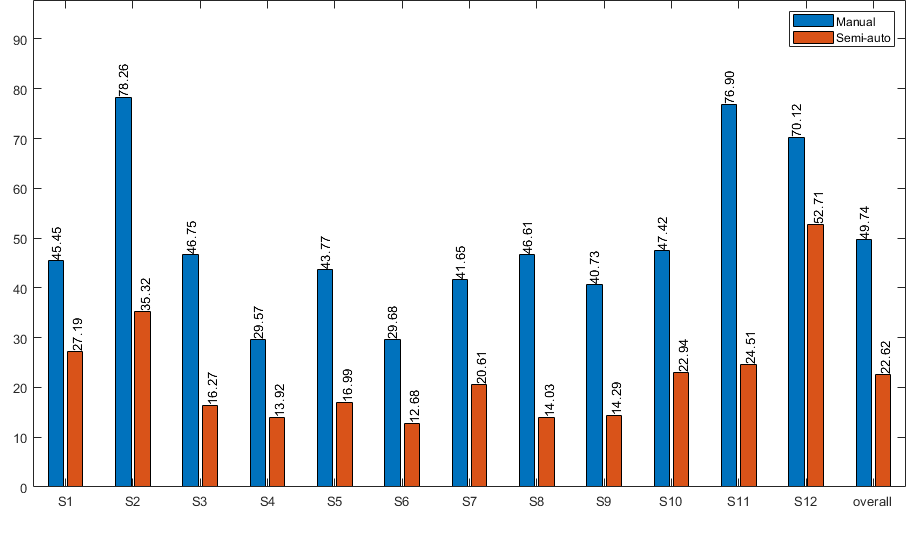


Table A3. Comparison of scoring time differences for detection of respiratory events between manual and semi-automatic scoring approaches at the recording level

| **Respiratory activity** | **n** | **Manual** | **Semi-Auto** | **Gain factor** | **Wilcoxon test *p*-value  (paired)** | **Effect size** |
| --- | --- | --- | --- | --- | --- | --- |
| SN1 | 12 | 18.02 [11.43, 16.97, 21.72, 43.78] | 11.26 [4.58, 8.68, 12.90, 41.26] | 1.60 | 0.0005* | 1.6927 |
| SN2 | 12 | 21.07 [10.79, 16.22, 32.51, 84.19] | 14.98 [5.90, 8.99, 20.34, 61.66] | 1.41 | 0.0342* | 0.7846 |
| SN3 | 12 | 56.56 [34.02, 51.09, 66.46, 119.81] | 23.50 [15.09, 18.11, 35.12, 74.93] | 2.41 | 0.0005* | 2.0932 |
| SN4 | 12 | 17.86 [9.59, 12.29, 27.44, 51.90] | 10.70 [4.87, 7.27, 13.04, 36.95] | 1.67 | 0.0005* | 1.3210 |
| SN5 | 12 | 27.26 [16.41, 20.28, 41.34, 104.06] | 17.19 [9.66, 14.04, 24.28, 55.81] | 1.59 | 0.0210* | 0.7914 |
| Overall | 60 | 23.81 [9.59, 17.62, 46.72, 119.81] | 14.58 [4.58, 10.46, 20.68, 74.93] | 1.63 | < 0.0001* | 0.9474 |

**Statistically significant result*

Figure A3. Comparison of individual scorer times for detection of respiratory events between manual and semi-automatic scoring approaches


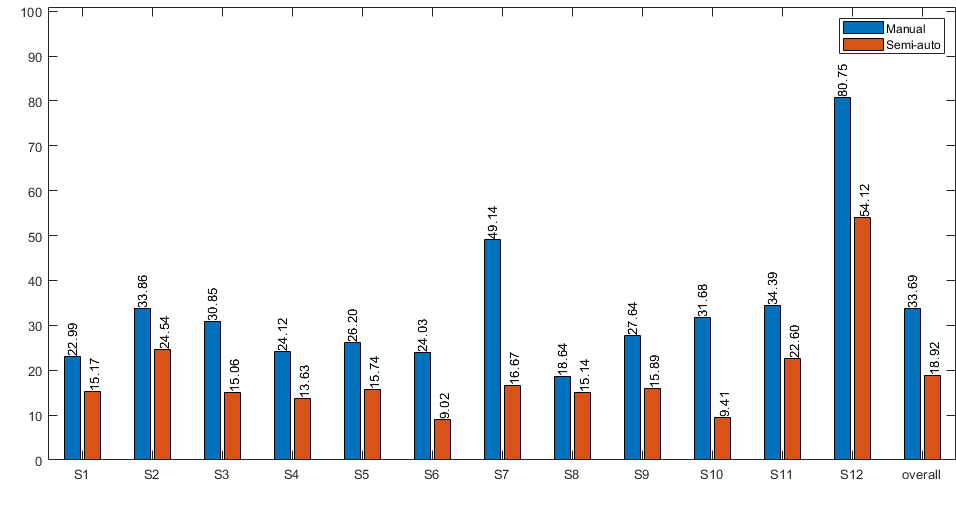


Table A4. Comparison of scoring time differences for EEG arousal detection between manual and semi-automatic scoring approaches at the recording level

| **EEG arousals** | **n** | **Manual** | **Semi-Auto** | **Gain factor** | **Wilcoxon test *p*-value  (paired)** | **Effect size** |
| --- | --- | --- | --- | --- | --- | --- |
| SN1 | 12 | 27.26 [16.59, 23.31, 40.51, 67.52] | 23.52 [15.33, 18.36, 26.70, 84.15] | 1.16 | 0.1294 | 0.5108 |
| SN2 | 12 | 20.66 [12.91, 17.77, 26.91, 48.25] | 17.49 [9.34, 12.84, 21.13, 66.78] | 1.18 | 0.1514 | 0.2718 |
| SN3 | 12 | 32.41 [18.75, 27.39, 38.13, 92.15] | 24.02 [5.06, 14.71, 37.49, 99.56] | 1.35 | 0.0425* | 0.7649 |
| SN4 | 12 | 27.81 [12.94, 17.40, 39.42, 69.77] | 19.55 [10.20, 15.26, 28.46, 91.92] | 1.42 | 0.2661 | 0.3343 |
| SN5 | 12 | 31.30 [20.05, 22.67, 42.37, 76.56] | 25.84 [12.27, 23.80, 35.22, 91.98] | 1.21 | 0.3013 | 0.2310 |
| Overall | 60 | 27.50 [12.91, 21.22, 37.65, 92.15] | 21.78 [5.06, 15.96, 28.59, 99.56] | 1.26 | 0.0011* | 0.4233 |

**Statistically significant result*

Figure A4. Comparison of individual scorer times for EEG arousal detection between manual and semi-automatic scoring approaches

**
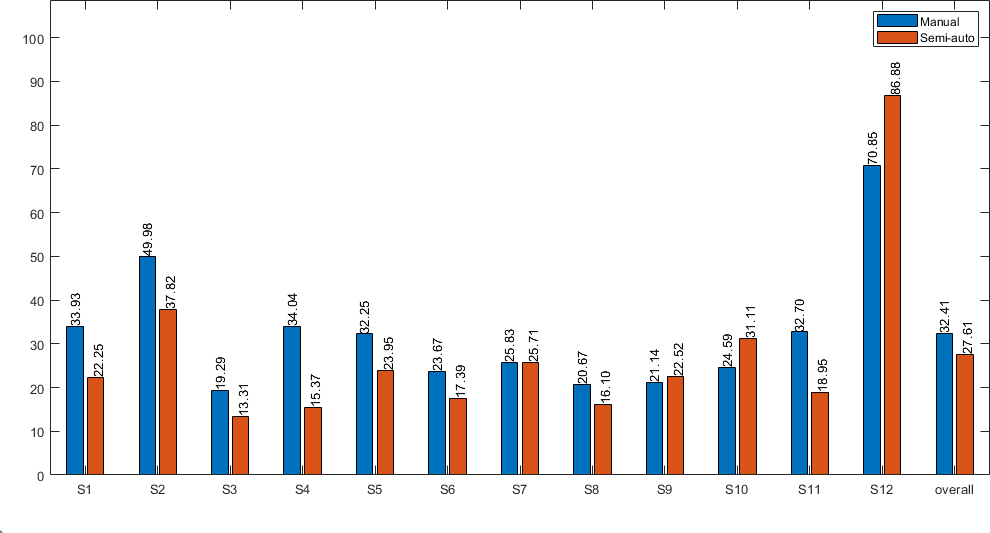
**
