# Supplementary material for: Computer-assisted analysis of polysomnographic recordings improves inter-scorer associated agreement and scoring times
Source: PLoS One. 2022 Sep 29;17(9):e0275530. doi: 10.1371/journal.pone.0275530 (PMC9522290; doi:10.1371/journal.pone.0275530)
Supplement: S2 Appendix — (DOCX) [file pone.0275530.s002.docx]

**S2 Appendix. Kappa-agreement individualized per-recording analyses**

Supporting information regarding main manuscript:

“**Computer-assisted analysis of polysomnographic recordings improves inter-scorer associated agreement and scoring times”**

Diego Alvarez-Estevez, Roselyne M. Rijsman

Common notation considerations: data distributions are characterized using the five-number summary as *p50 [minimum, p25, p75, maximum]*, where *XX* in the *pXX* notation refers to the corresponding percentile value. For further details, see Methods section in the main manuscript.

Table B1. Comparison of inter-scorer kappa agreement for the sleep staging task between manual and semi-automatic scoring approaches at the recording level

| **Sleep stages (TIB)** | **n** | **Value a priori**** | **Paired agreement distribution summary descriptors** | | **Wilcoxon test *p*-value  (paired)** | **Effect size** |
| --- | --- | --- | --- | --- | --- | --- |
|  |  |  | Manual | Semi-Auto |  |  |
| SN1 | 66 | 50^th^ / 0.74 | 0.75 [0.47, 0.70, 0.80, 0.86] | 0.80 [0.70, 0.78, 0.82, 0.88] | < 0.0001* | -0.6600 |
| SN2 | 66 | 62.5^th^ / 0.77 | 0.66 [0.36, 0.62, 0.72, 0.83] | 0.77 [0.53, 0.72, 0.80, 0.93] | < 0.0001* | -1.2949 |
| SN3 | 66 | 12.5^th^ / 0.58 | 0.75 [0.52, 0.70, 0.78, 0.83] | 0.79 [0.64, 0.74, 0.82, 0.90] | < 0.0001* | -1.0604 |
| SN4 | 66 | 37.5^th^ / 0.70 | 0.77 [0.65, 0.74, 0.80, 0.83] | 0.82 [0.64, 0.78, 0.85, 0.94] | < 0.0001* | -0.5528 |
| SN5 | 66 | 87.5^th^ / 0.83 | 0.80 [0.62, 0.77, 0.84, 0.89] | 0.84 [0.69, 0.79, 0.87, 0.92] | 0.0015* | -0.4578 |
| Overall | 330 | --- | 0.76 [0.36, 0.69, 0.80, 0.89] | 0.80 [0.53, 0.76, 0.83, 0.94] | < 0.0001* | -0.7146 |

**Statistically significant result
**Value a priori: Distribution percentile and corresponding kappa agreement obtained during the selection procedure between the automatic algorithm and the retrospective expert scorings in the clinical pre-sample database (see Methods section)*

Table B2. Comparison of inter-scorer kappa agreement for the leg movements’ detection task between manual and semi-automatic scoring approaches at the recording level

| **Limb movements (TIB)** | **n** | **Value a priori**** | **Paired agreement distribution summary descriptors** | | **Wilcoxon test *p*-value  (paired)** | **Effect size** |
| --- | --- | --- | --- | --- | --- | --- |
|  |  |  | Manual | Semi-Auto |  |  |
| SN1 | 66 | 50^th^ / 0.96 | 0.62 [0.46, 0.56, 0.69, 0.82] | 0.87 [0.77, 0.85, 0.93, 0.97] | < 0.0001* | -2.8314 |
| SN2 | 66 | 37.5^th^ / 0.95 | 0.71 [0.45, 0.64, 0.75, 0.87] | 0.91 [0.79, 0.87, 0.93, 0.98] | < 0.0001* | -2.5934 |
| SN3 | 66 | 62.5^th^ / 0.98 | 0.78 [0.57, 0.67, 0.84, 0.92] | 0.96 [0.79, 0.94, 0.97, 0.99] | < 0.0001* | -1.7358 |
| SN4 | 66 | 87.5^th^ / 1.00 | 0.79 [0.67, 0.75, 0.82, 0.91] | 0.94 [0.82, 0.91, 0.96, 0.98] | < 0.0001* | -2.5309 |
| SN5 | 66 | 12.5^th^ / 0.86 | 0.71 [0.47, 0.62, 0.77, 0.86] | 0.86 [0.78, 0.84, 0.89, 0.94] | < 0.0001* | -1.7866 |
| Overall | 330 | --- | 0.72 [0.45, 0.64, 0.79, 0.92] | 0.91 [0.77, 0.86, 0.95, 0.99] | < 0.0001* | -2.0223 |

**Statistically significant result
**Value a priori: Distribution percentile and corresponding kappa agreement obtained during the selection procedure between the automatic algorithm and the retrospective expert scorings in the clinical pre-sample database (see Methods section)*

Table B3. Comparison of inter-scorer kappa agreement for the leg movements’ detection task in wakefulness between manual and semi-automatic scoring approaches at the recording level

| **Limb movements (Wake)** | **n** | **Paired agreement distribution summary descriptors** | | **Wilcoxon test *p*-value  (paired)** | **Effect size** |
| --- | --- | --- | --- | --- | --- |
|  |  | Manual | Semi-Auto |  |  |
| SN1 | 66 | 0.52 [0.28, 0.45, 0.60, 0.78] | 0.82 [0.66, 0.79, 0.91, 0.97] | < 0.0001* | -2.4747 |
| SN2 | 66 | 0.55 [0.19, 0.40, 0.65, 0.82] | 0.84 [0.66, 0.78, 0.89, 0.99] | < 0.0001* | -1.7473 |
| SN3 | 66 | 0.78 [0.57, 0.69, 0.83, 0.91] | 0.97 [0.78, 0.95, 0.98, 0.99] | < 0.0001* | -1.7562 |
| SN4 | 66 | 0.73 [0.54, 0.66, 0.79, 0.90] | 0.92 [0.69, 0.88, 0.96, 0.99] | < 0.0001* | -1.9417 |
| SN5 | 66 | 0.72 [0.51, 0.65, 0.78, 0.87] | 0.89 [0.79, 0.86, 0.92, 0.95] | < 0.0001* | -2.3142 |
| Overall | 330 | 0.67 [0.19, 0.57, 0.77, 0.91] | 0.89 [0.66, 0.82, 0.94, 0.99] | < 0.0001* | -1.7121 |

**Statistically significant result*

Table B4. Comparison of inter-scorer kappa agreement for the leg movements’ detection task in sleep between manual and semi-automatic scoring approaches at the recording level

| **Limb movements (TST)** | **n** | **Paired agreement distribution summary descriptors** | | **Wilcoxon test *p*-value  (paired)** | **Effect size** |
| --- | --- | --- | --- | --- | --- |
|  |  | Manual | Semi-Auto |  |  |
| SN1 | 66 | 0.70 [0.49, 0.62, 0.75, 0.83] | 0.92 [0.80, 0.89, 0.94, 0.97] | < 0.0001* | -2.6375 |
| SN2 | 66 | 0.75 [0.49, 0.67, 0.80, 0.88] | 0.92 [0.80, 0.89, 0.94, 0.99] | < 0.0001* | -2.4712 |
| SN3 | 66 | 0.76 [0.53, 0.62, 0.83, 0.92] | 0.95 [0.77, 0.91, 0.96, 0.99] | < 0.0001* | -1.6278 |
| SN4 | 66 | 0.82 [0.73, 0.78, 0.85, 0.92] | 0.95 [0.88, 0.94, 0.96, 0.98] | < 0.0001* | -2.7504 |
| SN5 | 66 | 0.70 [0.39, 0.53, 0.77, 0.89] | 0.82 [0.66, 0.78, 0.85, 0.93] | < 0.0001* | -1.0254 |
| Overall | 330 | 0.75 [0.39, 0.65, 0.81, 0.92] | 0.92 [0.66, 0.86, 0.95, 0.99] | < 0.0001* | -1.6704 |

**Statistically significant result*

Table B5. Comparison of inter-scorer kappa agreement for the scoring of respiratory events between manual and semi-automatic scoring approaches at the recording level

| **Respiratory Events (Apnea, Hypopnea, RERA)  (TIB)** | **n** | **Value a priori**** | **Paired agreement distribution summary descriptors** | | **Wilcoxon test *p*-value  (paired)** | **Effect size** |
| --- | --- | --- | --- | --- | --- | --- |
|  |  |  | Manual | Semi-Auto |  |  |
| SN1 | 66 | 62.5^th^ / 0.86 | 0.75 [0.66, 0.71, 0.78, 0.86] | 0.86 [0.75, 0.84, 0.89, 0.94] | < 0.0001* | -1.7838 |
| SN2 | 66 | 12.5^th^ / 0.37 | 0.47 [0.22, 0.34, 0.52, 0.68] | 0.51 [0.30, 0.43, 0.60, 0.77] | 0.0021* | -0.4249 |
| SN3 | 66 | 87.5^th^ / 0.88 | 0.85 [0.72, 0.82, 0.87, 0.92] | 0.92 [0.88, 0.91, 0.94, 0.95] | < 0.0001* | -2.3013 |
| SN4 | 66 | 37.5^th^ / 0.65 | 0.46 [0.06, 0.37, 0.53, 0.68] | 0.47 [0.20, 0.40, 0.59, 0.78] | 0.0019* | -0.4262 |
| SN5 | 66 | 50^th^ / 0.68 | 0.47 [0.18, 0.38, 0.53, 0.62] | 0.64 [0.47, 0.59, 0.68, 0.80] | < 0.0001* | -1.9555 |
| Overall | 330 | --- | 0.55 [0.06, 0.43, 0.78, 0.92] | 0.66 [0.20, 0.53, 0.89, 0.95] | < 0.0001* | -0.8315 |

**Statistically significant result
**Value a priori: Distribution percentile and corresponding kappa agreement obtained during the selection procedure between the automatic algorithm and the retrospective expert scorings in the clinical pre-sample database (see Methods section)*

Table B6. Comparison of inter-scorer kappa agreement for the scoring of respiratory events (apneas only) between manual and semi-automatic scoring approaches at the recording level

| **Respiratory Events (Apneas only)  (TIB)** | **n** | **Paired agreement distribution summary descriptors** | | **Wilcoxon test *p*-value  (paired)** | **Effect size** |
| --- | --- | --- | --- | --- | --- |
|  |  | Manual | Semi-Auto |  |  |
| SN1 | 66 | 0.75 [0.56, 0.70, 0.79, 0.92] | 0.85 [0.72, 0.82, 0.94, 1.00] | < 0.0001* | -1.0425 |
| SN2 | 66 | 0.33 [-0.00, 0.00, 0.60, 0.77] | 0.00 [-0.00, 0.00, 0.41, 1.00] | 0.2374 | 0.1293 |
| SN3 | 66 | 0.86 [0.75, 0.84, 0.88, 0.93] | 0.96 [0.91, 0.95, 0.96, 0.98] | < 0.0001* | -2.7070 |
| SN4 | 66 | 1.00 [0.00, 1.00, 1.00, 1.00] | 1.00 [1.00, 1.00, 1.00, 1.00] | 0.0010* | -0.4438 |
| SN5 | 66 | 0.32 [-0.00, 0.23, 0.44, 0.65] | 0.58 [0.33, 0.48, 0.65, 0.82] | < 0.0001* | -1.1821 |
| Overall | 330 | 0.74 [-0.00, 0.35, 0.88, 1.00] | 0.88 [-0.00, 0.57, 0.98, 1.00] | < 0.0001* | -0.3783 |

**Statistically significant result*

Table B7. Comparison of inter-scorer kappa agreement for the scoring of respiratory events (hypopneas only) between manual and semi-automatic scoring approaches at the recording level

| **Respiratory Events (Hypopneas only)  (TIB)** | **n** | **Paired agreement distribution summary descriptors** | | **Wilcoxon test *p*-value  (paired)** | **Effect size** |
| --- | --- | --- | --- | --- | --- |
|  |  | Manual | Semi-Auto |  |  |
| SN1 | 66 | 0.51 [0.29, 0.42, 0.59, 0.80] | 0.75 [0.59, 0.71, 0.81, 0.90] | < 0.0001* | -1.7069 |
| SN2 | 66 | 0.44 [0.16, 0.30, 0.53, 0.71] | 0.52 [0.31, 0.44, 0.62, 0.79] | 0.0001* | -0.5570 |
| SN3 | 66 | 0.44 [0.15, 0.35, 0.52, 0.72] | 0.58 [0.38, 0.55, 0.63, 0.72] | < 0.0001* | -1.1295 |
| SN4 | 66 | 0.46 [0.06, 0.37, 0.54, 0.68] | 0.47 [0.20, 0.40, 0.59, 0.78] | 0.0021* | -0.4231 |
| SN5 | 66 | 0.46 [0.14, 0.34, 0.52, 0.60] | 0.63 [0.48, 0.59, 0.67, 0.81] | < 0.0001* | -1.8539 |
| Overall | 330 | 0.46 [0.06, 0.36, 0.53, 0.80] | 0.61 [0.20, 0.51, 0.68, 0.90] | < 0.0001* | -0.9569 |

**Statistically significant result*

Table B8. Comparison of inter-scorer kappa agreement for the EEG arousals’ scoring task between manual and semi-automatic scoring approaches at the recording level

| **EEG Arousals (TIB)** | **n** | **Value a priori**** | **Paired agreement distribution summary descriptors** | | **Wilcoxon test *p*-value  (paired)** | **Effect size** |
| --- | --- | --- | --- | --- | --- | --- |
|  |  |  | Manual | Semi-Auto |  |  |
| SN1 | 66 | 12.5^th^ / 0.30 | 0.54 [0.40, 0.48, 0.59, 0.66] | 0.54 [0.38, 0.50, 0.57, 0.65] | 0.6944 | 0.0360 |
| SN2 | 66 | 37.5^th^ / 0.51 | 0.48 [0.19, 0.41, 0.54, 0.67] | 0.56 [0.41, 0.52, 0.62, 0.67] | < 0.0001* | -1.0501 |
| SN3 | 66 | 50^th^ / 0.56 | 0.60 [0.38, 0.56, 0.64, 0.71] | 0.66 [0.50, 0.62, 0.69, 0.87] | < 0.0001* | -0.8658 |
| SN4 | 66 | 67.5^th^ / 0.62 | 0.67 [0.38, 0.62, 0.72, 0.80] | 0.73 [0.59, 0.67, 0.76, 0.82] | 0.0005* | -0.5154 |
| SN5 | 66 | 87.5^th^ - 0.81 | 0.64 [0.33, 0.52, 0.70, 0.79] | 0.71 [0.57, 0.69, 0.74, 0.81] | < 0.0001* | -0.7811 |
| Overall | 330 | --- | 0.58 [0.19, 0.48, 0.65, 0.80] | 0.65 [0.38, 0.56, 0.71, 0.87] | < 0.0001* | -0.6166 |

**Statistically significant result
**Value a priori: Distribution percentile and corresponding kappa agreement obtained during the selection procedure between the automatic algorithm and the retrospective expert scorings in the clinical pre-sample database (see Methods section of main manuscript)*
