# Supplementary material for: Computer-assisted analysis of polysomnographic recordings improves inter-scorer associated agreement and scoring times
Source: PLoS One. 2022 Sep 29;17(9):e0275530. doi: 10.1371/journal.pone.0275530 (PMC9522290; doi:10.1371/journal.pone.0275530)
Supplement: S3 Appendix — (DOCX) [file pone.0275530.s003.docx]

**S3 Appendix. Diagnostic-indices individualized inter-scorer variability per-recording analyses**

Supporting information regarding main manuscript:

“**Computer-assisted analysis of polysomnographic recordings improves inter-scorer associated agreement and scoring times”**

Diego Alvarez-Estevez, Roselyne M. Rijsman

Common notation considerations: data distributions are characterized using the five-number summary as *p50 [minimum, p25, p75, maximum]*, where *XX* in the *pXX* notation refers to the corresponding percentile value. For further details, see Methods section in the main manuscript.

Table C1. Comparison of derived diagnostic indices for Sleep Efficiency between manual and semi-automatic scoring approaches at the recording level

| **Sleep Efficiency (SE)** | **n** | **Summary of distributions** | | **Wilcoxon test *p*-value  (paired)** | **Effect size** | **Normalized standard deviation** | | **Brown-Forsythe *p*-value (unpaired)** | **Variance Ratio**** |
| --- | --- | --- | --- | --- | --- | --- | --- | --- | --- |
|  |  | Manual | Semi-Auto |  |  | Manual | Semi-auto |  |  |
| SN1 | 12 | 94.75 [87.00, 93.19, 95.19, 96.37] | 93.75 [92.57, 92.91, 94.86, 96.03] | 0.7485 | -0.0293 | 0.03 | 0.01 | 0.4720 | 4.2720 |
| SN2 | 12 | 95.82 [93.48, 95.41, 96.78, 98.03] | 96.51 [95.54, 95.89, 97.13, 97.61] | 0.1133 | -0.4941 | 0.01 | 0.01 | 0.0925 | 3.6102 |
| SN3 | 12 | 89.45 [87.56, 88.23, 90.22, 92.26] | 90.31 [89.63, 89.82, 90.73, 91.29] | 0.0771 | -0.5589 | 0.02 | 0.01 | 0.0099* | 7.6163 |
| SN4 | 12 | 69.29 [66.17, 68.05, 71.90, 74.08] | 71.12 [67.01, 70.59, 71.90, 76.37] | 0.0269* | -0.6550 | 0.04 | 0.03 | 0.1596 | 1.6385 |
| SN5 | 12 | 24.09 [22.79, 23.24, 25.61, 34.41] | 26.45 [23.24, 25.55, 27.81, 31.25] | 0.0791 | -0.4044 | 0.13 | 0.08 | 0.6156 | 2.5403 |
| Overall | 60 | 89.11 [22.79, 68.05, 94.87, 98.03] | 90.31 [23.24, 70.59, 94.86, 97.61] | 0.0004* | -0.3717 | 0.06 | 0.04 | 0.1384 | 2.5018 |

**Statistically significant result
**Calculated as Var[Manual]/Var[Semi-Auto], thus values > 1 point out toward higher dispersion (variability) among the manual scoring distribution*

Table C2. Comparison of derived diagnostic indices for Sleep Onset Latency between manual and semi-automatic scoring approaches at the recording level

| **Sleep Onset Latency (SOL)** | **n** | **Summary of distributions** | | **Wilcoxon test *p*-value  (paired)** | **Effect size** | **Normalized standard deviation** | | **Brown-Forsythe *p*-value (unpaired)** | **Variance Ratio**** |
| --- | --- | --- | --- | --- | --- | --- | --- | --- | --- |
|  |  | Manual | Semi-Auto |  |  | Manual | Semi-auto |  |  |
| SN1 | 12 | 0.63 [0.63, 0.63, 3.38, 5.13] | 1.88 [0.13, 0.63, 2.88, 11.13] | 0.5898 | -0.2672 | 0.92 | 1.20 | 0.9128 | 0.5898 |
| SN2 | 12 | 3.00 [1.50, 2.75, 5.00, 5.50] | 2.50 [2.50, 2.50, 3.00, 4.50] | 0.1367 | 0.5499 | 0.37 | 0.20 | 0.1675 | 3.2171 |
| SN3 | 12 | 3.21 [0.00, 0.71, 6.71, 7.21] | 1.96 [0.00, 0.71, 6.21, 6.21] | 0.5342 | 0.1651 | 0.81 | 0.93 | 0.5356 | 0.7597 |
| SN4 | 12 | 106.91 [5.41, 15.66, 107.41, 107.41] | 106.41 [1.91, 16.91, 106.91, 107.41] | 0.5938 | -0.0526 | 0.63 | 0.62 | 0.9670 | 1.0401 |
| SN5 | 12 | 168.20 [73.20, 148.45, 169.70, 169.70] | 112.95 [103.20, 103.20, 168.20, 169.70] | 0.1230 | 0.5270 | 0.20 | 0.24 | 0.3818 | 0.7176 |
| Overall | 60 | 5.27 [0.00, 2.50, 107.41, 169.70] | 4.11 [0.00, 2.31, 104.81, 169.70] | 0.0629 | 0.2227 | 0.62 | 0.72 | 0.8598 | 0.7455 |

**Statistically significant result
**Calculated as Var[Manual]/Var[Semi-Auto], thus values > 1 point out toward higher dispersion (variability) among the manual scoring distribution*

Table C3. Comparison of derived diagnostic indices for Wake After Sleep Onset between manual and semi-automatic scoring approaches at the recording level

| **Wake After Sleep Onset (WASO)** | **n** | **Summary of distributions** | | **Wilcoxon test *p*-value  (paired)** | **Effect size** | **Normalized standard deviation** | | **Brown-Forsythe *p*-value (unpaired)** | **Variance Ratio**** |
| --- | --- | --- | --- | --- | --- | --- | --- | --- | --- |
|  |  | Manual | Semi-Auto |  |  | Manual | Semi-auto |  |  |
| SN1 | 12 | 23.53 [16.28, 21.52, 30.46, 58.28] | 28.02 [17.78, 23.02, 31.75, 33.24] | 0.7334 | 0.0303* | 0.40 | 0.19 | 0.4846 | 4.1972 |
| SN2 | 12 | 15.09 [7.12, 11.59, 16.61, 23.58] | 12.61 [8.62, 10.36, 14.84, 16.12] | 0.1050 | 0.4941 | 0.31 | 0.19 | 0.1804 | 2.6980 |
| SN3 | 12 | 86.20 [63.25, 79.95, 96.15, 101.74] | 79.18 [71.25, 75.70, 83.23, 84.76] | 0.0771 | 0.5588 | 0.14 | 0.06 | 0.0152* | 6.3587 |
| SN4 | 12 | 145.76 [124.46, 134.84, 151.77, 160.76] | 137.03 [113.31, 134.03, 139.51, 156.76] | 0.0269 | 0.6577 | 0.08 | 0.07 | 0.2223 | 1.3734 |
| SN5 | 12 | 333.66 [289.49, 327.69, 337.39, 339.39] | 323.62 [302.99, 318.24, 327.62, 337.38] | 0.0640 | 0.3964 | 0.04 | 0.03 | 0.6838 | 2.2134 |
| Overall | 60 | 86.20 [7.12, 22.27, 151.77, 339.39] | 79.18 [8.62, 23.02, 139.51, 337.38] | 0.0003 | 0.3929 | 0.23 | 0.12 | 0.0307* | 3.4201 |

**Statistically significant result
**Calculated as Var[Manual]/Var[Semi-Auto], thus values > 1 point out toward higher dispersion (variability) among the manual scoring distribution*

Table C4. Comparison of derived diagnostic indices for Limb Movement Index (LMI) between manual and semi-automatic scoring approaches at the recording level

| **Limb Movement Index (LMI)** | **n** | **Summary of distributions** | | **Wilcoxon test *p*-value  (paired)** | **Effect size** | **Normalized standard deviation** | | **Brown-Forsythe *p*-value (unpaired)** | **Variance Ratio**** |
| --- | --- | --- | --- | --- | --- | --- | --- | --- | --- |
|  |  | Manual | Semi-Auto |  |  | Manual | Semi-auto |  |  |
| SN1 | 12 | 25.94 [23.01, 23.54, 30.98, 35.79] | 31.73 [29.62, 30.30, 32.48, 35.19] | 0.0122* | -0.9204 | 0.17 | 0.05 | 0.0164* | 11.0770 |
| SN2 | 12 | 19.78 [14.28, 17.92, 22.38, 25.28] | 21.27 [20.52, 20.89, 22.46, 23.65] | 0.0732 | -0.6051 | 0.16 | 0.05 | 0.0098* | 12.3865 |
| SN3 | 12 | 17.64 [14.87, 15.61, 21.06, 22.02] | 19.18 [17.21, 17.93, 19.68, 20.27] | 0.2915 | -0.2779 | 0.15 | 0.05 | 0.0013* | 8.2051 |
| SN4 | 12 | 104.81 [91.66, 101.25, 111.77, 116.78] | 107.69 [100.32, 105.32, 108.97, 115.25] | 0.5059 | -0.2701 | 0.08 | 0.03 | 0.0317* | 4.8308 |
| SN5 | 12 | 59.52 [16.24, 40.61, 70.19, 81.94] | 50.42 [32.00, 40.48, 61.82, 67.88] | 0.4316 | 0.1816 | 0.39 | 0.24 | 0.2112 | 2.6541 |
| Overall | 60 | 25.27 [14.28, 19.78, 70.19, 116.78] | 31.73 [17.21, 20.89, 61.82, 115.25] | 0.0999 | -0.0763 | 0.21 | 0.11 | 0.0003* | 3.5592 |

**Statistically significant result
**Calculated as Var[Manual]/Var[Semi-Auto], thus values > 1 point out toward higher dispersion (variability) among the manual scoring distribution*

Table C5. Comparison of derived diagnostic indices for Periodic Limb Movement Index (PLMI) between manual and semi-automatic scoring approaches at the recording level

| **Periodic Limb Movement Index (PLMI)** | **n** | **Summary of distributions** | | **Wilcoxon test *p*-value  (paired)** | **Effect size** | **Normalized standard deviation** | | **Brown-Forsythe *p*-value (unpaired)** | **Variance Ratio**** |
| --- | --- | --- | --- | --- | --- | --- | --- | --- | --- |
|  |  | Manual | Semi-Auto |  |  | Manual | Semi-auto |  |  |
| SN1 | 12 | 12.41 [8.57, 10.16, 15.11, 20.60] | 13.84 [12.03, 13.46, 14.52, 16.84] | 0.2036 | -0.2777 | 0.30 | 0.10 | 0.0038* | 9.1267 |
| SN2 | 12 | 12.79 [6.99, 10.41, 13.75, 14.72] | 13.32 [12.49, 12.94, 13.46, 15.32] | 0.0771 | -0.6003 | 0.21 | 0.05 | 0.0052* | 15.5409 |
| SN3 | 12 | 1.46 [0.58, 0.87, 2.77, 4.37] | 2.48 [1.75, 2.41, 3.28, 4.08] | 0.0791 | -0.6395 | 0.65 | 0.23 | 0.0144* | 7.7557 |
| SN4 | 12 | 93.70 [83.17, 91.49, 99.72, 103.37] | 96.92 [89.11, 95.98, 97.94, 102.01] | 0.3203 | -0.3522 | 0.06 | 0.03 | 0.0589 | 3.7146 |
| SN5 | 12 | 52.97 [11.39, 31.16, 61.70, 71.27] | 42.06 [22.30, 32.12, 53.70, 61.09] | 0.3013 | 0.1796 | 0.45 | 0.29 | 0.3426 | 2.3274 |
| Overall | 60 | 13.91 [0.58, 9.20, 61.70, 103.37] | 14.14 [1.75, 12.79, 53.70, 102.01] | 0.1842 | -0.0245 | 0.38 | 0.11 | < 0.0001* | 4.9196 |

**Statistically significant result
**Calculated as Var[Manual]/Var[Semi-Auto], thus values > 1 point out toward higher dispersion (variability) among the manual scoring distribution*

Table C6. Comparison of derived diagnostic indices for Apnea-Hypopnea Index (AHI) between manual and semi-automatic scoring approaches at the recording level

| **Apnea-Hypopnea Index (AHI)** | **n** | **Summary of distributions** | | **Wilcoxon test *p*-value  (paired)** | **Effect size** | **Normalized standard deviation** | | **Brown-Forsythe *p*-value (unpaired)** | **Variance Ratio**** |
| --- | --- | --- | --- | --- | --- | --- | --- | --- | --- |
|  |  | Manual | Semi-Auto |  |  | Manual | Semi-auto |  |  |
| SN1 | 12 | 5.96 [3.97, 5.09, 6.39, 6.56] | 6.21 [3.45, 5.53, 6.56, 6.91] | 0.2915 | -0.1499 | 0.15 | 0.18 | 0.9174 | 0.7009 |
| SN2 | 12 | 3.71 [0.21, 1.75, 5.67, 6.60] | 4.74 [1.03, 4.54, 6.19, 7.84] | 0.2256 | -0.3764 | 0.56 | 0.39 | 0.1495 | 1.9995 |
| SN3 | 12 | 53.73 [44.73, 51.75, 54.31, 55.79] | 56.20 [53.81, 55.38, 57.03, 58.10] | 0.0010* | -1.0041 | 0.05 | 0.02 | 0.3391 | 5.1273 |
| SN4 | 12 | 3.49 [0.17, 2.58, 4.08, 5.65] | 4.49 [1.99, 2.66, 5.49, 7.15] | 0.1685 | -0.4898 | 0.43 | 0.40 | 0.8031 | 1.1386 |
| SN5 | 12 | 9.47 [3.56, 6.20, 12.47, 14.25] | 13.32 [7.98, 10.54, 14.61, 17.67] | 0.0098* | -0.9169 | 0.37 | 0.24 | 0.0945 | 2.4070 |
| Overall | 60 | 5.86 [0.17, 3.71, 12.47, 55.79] | 6.54 [1.03, 4.70, 14.61, 58.10] | < 0.0001* | -0.0906 | 0.35 | 0.28 | 0.2058 | 1.6157 |

**Statistically significant result
**Calculated as Var[Manual]/Var[Semi-Auto], thus values > 1 point out toward higher dispersion (variability) among the manual scoring distribution*

Table C7. Comparison of derived diagnostic indices for Apnea Index (AI) between manual and semi-automatic scoring approaches at the recording level

| **Apnea Index (AI)** | **n** | **Summary of distributions** | | **Wilcoxon test *p*-value  (paired)** | **Effect size** | **Normalized standard deviation** | | **Brown-Forsythe *p*-value (unpaired)** | **Variance Ratio**** |
| --- | --- | --- | --- | --- | --- | --- | --- | --- | --- |
|  |  | Manual | Semi-Auto |  |  | Manual | Semi-auto |  |  |
| SN1 | 12 | 3.11 [2.24, 2.76, 3.46, 5.01] | 3.02 [2.59, 2.76, 3.54, 3.80] | 0.6797 | 0.1934 | 0.23 | 0.13 | 0.4082 | 3.1926 |
| SN2 | 12 | 0.72 [0.00, 0.31, 1.03, 1.44] | 0.41 [0.00, 0.00, 0.62, 1.24] | 0.2500 | 0.4379 | 0.63 | 1.07 | 0.1241 | 0.3393 |
| SN3 | 12 | 50.59 [42.75, 49.11, 51.83, 52.49] | 51.50 [48.86, 50.09, 53.14, 53.48] | 0.0332* | -0.6133 | 0.06 | 0.03 | 0.4382 | 2.8018 |
| SN4 | 12 | 0.00 [0.00, 0.00, 0.00, 0.17] | 0.00 [0.00, 0.00, 0.00, 0.00] | 1.0000 | 0.2887 | 3.46 | 0.00 | 0.3282 | --- |
| SN5 | 12 | 0.92 [0.43, 0.50, 1.57, 2.71] | 1.57 [0.86, 1.43, 2.07, 2.71] | 0.0439* | -0.7011 | 0.66 | 0.34 | 0.0423* | 3.6581 |
| Overall | 60 | 1.26 [0.00, 0.31, 3.46, 52.49] | 1.57 [0.00, 0.00, 3.54, 53.48] | 0.0840 | -0.2483 | 1.55 | 0.64 | 0.3685 | 5.9561 |

**Statistically significant result
**Calculated as Var[Manual]/Var[Semi-Auto], thus values > 1 point out toward higher dispersion (variability) among the manual scoring distribution*

Table C8. Comparison of derived diagnostic indices for Hypopnea Index (HI) between manual and semi-automatic scoring approaches at the recording level

| **Hypopnea Index (HI)** | **n** | **Summary of distributions** | | **Wilcoxon test *p*-value  (paired)** | **Effect size** | **Normalized standard deviation** | | **Brown-Forsythe *p*-value (unpaired)** | **Variance Ratio**** |
| --- | --- | --- | --- | --- | --- | --- | --- | --- | --- |
|  |  | Manual | Semi-Auto |  |  | Manual | Semi-auto |  |  |
| SN1 | 12 | 2.42 [0.69, 2.16, 2.93, 3.28] | 2.76 [0.35, 2.42, 3.62, 4.14] | 0.2729 | -0.2322 | 0.32 | 0.44 | 0.3838 | 0.5168 |
| SN2 | 12 | 2.99 [0.21, 1.23, 4.75, 5.16] | 4.54 [1.03, 3.92, 5.57, 7.22] | 0.1050 | -0.5410 | 0.61 | 0.40 | 0.0563 | 2.3194 |
| SN3 | 12 | 2.73 [1.82, 2.31, 3.14, 4.79] | 4.71 [2.31, 3.71, 5.70, 6.77] | 0.0024* | -1.2402 | 0.29 | 0.28 | 0.7957 | 1.1134 |
| SN4 | 12 | 3.41 [0.17, 2.58, 4.08, 5.65] | 4.49 [1.99, 2.66, 5.49, 7.15] | 0.1685 | -0.4933 | 0.43 | 0.40 | 0.8395 | 1.1477 |
| SN5 | 12 | 7.70 [2.28, 5.78, 11.33, 13.54] | 11.12 [7.13, 8.91, 13.12, 15.68] | 0.0093* | -0.8548 | 0.43 | 0.25 | 0.0791 | 2.8708 |
| Overall | 60 | 3.10 [0.17, 2.38, 4.87, 13.54] | 4.55 [0.35, 3.13, 6.68, 15.68] | < 0.0001* | -0.6271 | 0.42 | 0.35 | 0.2702 | 1.4050 |

**Statistically significant result
**Calculated as Var[Manual]/Var[Semi-Auto], thus values > 1 point out toward higher dispersion (variability) among the manual scoring distribution*

Table C9. Comparison of derived diagnostic indices for Oxygen Desaturation Index (ODI) between manual and semi-automatic scoring approaches at the recording level

| **Oxygen Desaturation Index (ODI)** | **n** | **Summary of distributions** | | **Wilcoxon test *p*-value  (paired)** | **Effect size** | **Normalized standard deviation** | | **Brown-Forsythe *p*-value (unpaired)** | **Variance Ratio**** |
| --- | --- | --- | --- | --- | --- | --- | --- | --- | --- |
|  |  | Manual | Semi-Auto |  |  | Manual | Semi-auto |  |  |
| SN1 | 12 | 6.04 [3.28, 5.00, 6.47, 6.91] | 6.13 [5.35, 5.62, 6.56, 7.25] | 0.1563 | -0.5125 | 0.20 | 0.11 | 0.0978 | 3.6811 |
| SN2 | 12 | 10.41 [2.47, 5.36, 12.58, 14.64] | 12.68 [7.42, 10.62, 13.61, 15.05] | 0.0220* | 0.7914 | 0.47 | 0.19 | 0.0053* | 6.0152 |
| SN3 | 12 | 49.02 [3.14, 45.97, 64.37, 72.30] | 64.62 [49.19, 64.21, 65.94, 68.50] | 0.0186* | -0.6892 | 0.36 | 0.10 | 0.0296* | 13.8934 |
| SN4 | 12 | 3.49 [0.33, 2.41, 4.24, 4.99] | 5.90 [3.49, 5.16, 5.98, 6.48] | 0.0024* | -1.2408 | 0.42 | 0.14 | 0.0097* | 8.6081 |
| SN5 | 12 | 12.18 [3.71, 7.70, 14.68, 16.25] | 14.89 [10.55, 14.11, 15.89, 17.81] | 0.0171* | -0.8690 | 0.38 | 0.13 | 0.0127* | 8.5842 |
| Overall | 60 | 6.48 [0.33, 4.24, 14.38, 72.30] | 12.04 [3.49, 6.01, 15.89, 68.50] | < 0.0001* | -0.4759 | 0.36 | 0.13 | < 0.0001* | 7.5395 |

**Statistically significant result
**Calculated as Var[Manual]/Var[Semi-Auto], thus values > 1 point out toward higher dispersion (variability) among the manual scoring distribution*

Table C10. Comparison of derived diagnostic indices for Arousal Index (ArI) between manual and semi-automatic scoring approaches at the recording level

| **Arousal Index (ArI)** | **n** | **Summary of distributions** | | **Wilcoxon test *p*-value  (paired)** | **Effect size** | **Normalized standard deviation** | | **Brown-Forsythe *p*-value (unpaired)** | **Variance Ratio**** |
| --- | --- | --- | --- | --- | --- | --- | --- | --- | --- |
|  |  | Manual | Semi-Auto |  |  | Manual | Semi-auto |  |  |
| SN1 | 12 | 24.24 [15.63, 19.43, 26.84, 36.06] | 22.27 [13.60, 17.83, 25.52, 32.12] | 0.2661 | 0.3671 | 0.27 | 0.24 | 0.7511 | 1.3309 |
| SN2 | 12 | 8.51 [3.84, 6.13, 11.52, 17.20] | 9.60 [5.38, 7.53, 11.21, 12.20] | 0.7910 | -0.1051 | 0.43 | 0.24 | 0.0830 | 3.2394 |
| SN3 | 12 | 18.01 [12.98, 15.85, 24.25, 29.78] | 19.18 [13.50, 17.48, 24.10, 32.56] | 0.7910 | -0.2315 | 0.28 | 0.26 | 0.8114 | 1.1390 |
| SN4 | 12 | 14.29 [10.12, 11.70, 15.32, 18.87] | 15.07 [12.95, 14.12, 17.87, 23.32] | 0.0063* | -0.9293 | 0.20 | 0.20 | 0.9421 | 0.9210 |
| SN5 | 12 | 27.70 [15.12, 24.61, 32.30, 35.33] | 31.05 [23.70, 26.97, 33.71, 36.37] | 0.0425* | -0.5423 | 0.20 | 0.14 | 0.4315 | 2.0931 |
| Overall | 60 | 17.57 [3.84, 12.98, 25.64, 36.06] | 18.38 [5.38, 13.82, 25.41, 36.37] | 0.1756 | -0.1866 | 0.28 | 0.21 | 0.1300 | 1.7129 |

**Statistically significant result
**Calculated as Var[Manual]/Var[Semi-Auto], thus values > 1 point out toward higher dispersion (variability) among the manual scoring distribution*
