# Supplementary material for: Computer-assisted analysis of polysomnographic recordings improves inter-scorer associated agreement and scoring times
Source: PLoS One. 2022 Sep 29;17(9):e0275530. doi: 10.1371/journal.pone.0275530 (PMC9522290; doi:10.1371/journal.pone.0275530)
Supplement: S4 Appendix — (DOCX) [file pone.0275530.s004.docx]

**S4 Appendix. Correlation analyses for automatic selection of PSG recordings involved in the study**

Supporting information regarding main manuscript:

“**Computer-assisted analysis of polysomnographic recordings improves inter-scorer associated agreement and scoring times”**

Diego Alvarez-Estevez, Roselyne M. Rijsman

The main text manuscript describes an automatic PSG selection procedure to avoid selection bias. There the hypothesis is stated that automatic scoring difficulty would be correlated with manual scoring difficulty: the lower (higher) the agreement between the automatic and the clinical reference, the lower (higher) the expected inter-rater agreement should be.

Table D1 shows the results of correlation analyses between the automatic-clinical kappa agreement scores obtained during the selection process, and the respective levels of inter-scorer agreement obtained after the rescoring process has been performed. Individual a priori and a posteriori agreement kappa’s involved in the analyses can be found in the corresponding tables in section B of the Supplementary Materials.

Table D1. Correlation between automatic-clinical selection agreement with respect to manual and semi-automatic achieved agreements during rescoring tasks (using the kappa statistic)

| **Scoring task** | **n** | **Selection vs Manual** | | **Selection vs Semi-auto** | |
| --- | --- | --- | --- | --- | --- |
|  |  | *r* | *p*-value | *r* | *p*-value |
| Sleep staging | 5 | 0.0549 | 0.9301 | 0.3601 | 0.5516 |
| Leg Movements | 5 | 0.4423 | 0.4557 | 0.8591 | 0.0621 |
| Respiratory events | 5 | 0.7946 | 0.1083 | 0.8590 | 0.0622 |
| EEG Arousals | 5 | 0.5947 | 0.2902 | 0.8752 | 0.0519 |
| Altogether | 20 | 0.6645 | 0.0014* | 0.8789 | < 0.0001* |

**Statistically significant result*

Data from Table D1 shows heterogeneous individual per-task correlations that do not reach statistically significant levels. This is possibly due to the low number of cases involved per task in the corresponding calculations (only 5). When aggregating data from all the scoring tasks together, however, significant correlation effects are noticed. This is likely due to the increased number of available data points (n=20), therefore supporting the selection hypothesis. In general, higher correlation values are obtained in the context of the semi-automatic scoring scenario (r = 0.8789 vs r = 0.6645 with respect to the manual rescoring approach). This result is partially expected, as scorings derived from the semi-automatic approach more likely resemble the output of the pure automatic scoring algorithm (no human intervention) used during the selection process.
