# Supplementary material for: Computer-assisted analysis of polysomnographic recordings improves inter-scorer associated agreement and scoring times
Source: PLoS One. 2022 Sep 29;17(9):e0275530. doi: 10.1371/journal.pone.0275530 (PMC9522290; doi:10.1371/journal.pone.0275530)
Supplement: Attachment — Submitted filename: response-to-reviewers.docx [file pone.0275530.s006.docx]

Dear Editor/Reviewers,

We would like to thank the editor and all the reviewers for their time and useful comments. Please find the corresponding replies in the table below. With the aim to improve readability, our answers are provided following each question/comment in blue color.

Diego Alvarez-Estevez
Corresponding author

Response to reviewers

JOURNAL EDITOR

Journal Requirements:

When submitting your revision, we need you to address these additional requirements.

1. Please ensure that your manuscript meets PLOS ONE's style requirements, including those for file naming. The PLOS ONE style templates can be found at

https://journals.plos.org/plosone/s/file?id=wjVg/PLOSOne_formatting_sample_main_body.pdf and

https://journals.plos.org/plosone/s/file?id=ba62/PLOSOne_formatting_sample_title_authors_affiliations.pdf

We thank the feedback of the editor. The manuscript has been entirely and carefully reviewed matching the style guidelines pointed out by the editor to our best.

2. Thank you for stating the following financial disclosure: 

"This study has been initiated at Haaglanden Medisch Centrum under project number 2019-073. The study has also been partially funded under project ED431H 2020/10 of Xunta de Galicia. Authors wish to acknowledge the support received from the Centro de Investigación de Galicia (CITIC), funded by Xunta de Galicia and the European Union (European Regional Development Fund-Galicia 2014-2020 Program), by grant ED431G 2019/01"

Please state what role the funders took in the study.  If the funders had no role, please state: ""The funders had no role in study design, data collection and analysis, decision to publish, or preparation of the manuscript."" 
If this statement is not correct you must amend it as needed. 
Please include this amended Role of Funder statement in your cover letter; we will change the online submission form on your behalf

Amended versions of “Financial Disclosure” and “Role of Funder” statements have been included in the new cover letter. Specifically:

Financial Disclosure:

“This study has been initiated at Haaglanden Medisch Centrum with a cost neutral policy under project number 2019-073. One of the authors (DAE) has received funding under project ED431H 2020/10 of Xunta de Galicia. Authors wish to acknowledge the support received from the Centro de Investigación de Galicia (CITIC), funded by Xunta de Galicia and the European Union (European Regional Development Fund-Galicia 2014-2020 Program), by grant ED431G 2019/01”

Role of Funder statement:

“The funders had no role in study design, data collection and analysis, decision to publish, or preparation of the manuscript”

3. Please include captions for your Supporting Information files at the end of your manuscript, and update any in-text citations to match accordingly. Please see our Supporting Information guidelines for more information: <http://journals.plos.org/plosone/s/supporting-information>.

We have reorganized the supporting materials and updated all in-text citations according to the referenced guidelines

REVIEWER 1

Reviewer #1: The authors investigated inter-scorer agreement with 12 “expert technician scorers” on 5 PSG each selected for sleep staging, EEG arousals, “respiratory activity” and “identification of leg movements”. They found that when semi-automatic or computer assisted sleep scoring was used, the time for sleep analysis was reduced and suggest this could help to reduce waiting times for sleep laboratories.
I have several critical comments. First of all, the idea is not novel, and multiple methods have been described to assist sleep scoring, and this is only another of hundreds of papers on this topic. Second, from a methodological standpoint, it seems to lack a certain rigorousness, this begins with the abstract, and goes on within the text.

We thank the critical feedback. We have introduced several modifications to the text and have done our best to address and clarify all the major concerns expressed by the reviewer.

One first clarification regards to possible misinterpretation of the main objective of the work, as expressed in the abstract:

“To investigate inter-scorer agreement and scoring time differences associated with visual and computer-assisted analysis of polysomnographic (PSG) recordings”

and the main conclusions:

“Computer-assisted analysis can improve inter-scorer agreement and scoring times associated with the review of PSG studies resulting in higher efficiency and overall quality in the diagnosis sleep disorders”

hence, not limiting to the report of reduced scoring time with semi-automatic scoring to help shortening waiting lists for sleep labs, but including as well analysis and comparison of inter-rater agreement under both manual and semi-automatic approaches.

We have introduced several changes throughout the manuscript to highlight the overall scope and reduce the focus on the mere reduction of the waiting lists.

Likewise, we believe it is important to remark that the aim of the work neither is describing just another of multiple methods for automatic sleep scoring. We are not reporting how well or bad full-automatic scoring behaves against a particular expert reference. Instead we pursue quantification and comparison of the different levels of agreement and scoring times among several human experts under the use of manual or semi-automatic scoring. Changes have been introduced as well in the text in this regard, also in relation to the more specific comments from the reviewer. Those changes are described in more detail throughout the corresponding following comments.

As with regard to novelty, we have rearranged the text providing a more structured discussion, hoping to improve both readability and focus on the different contributions from our study. A summary of the main novelties and new results (addressed with more detail in the discussion) is also provided next:

To the best of our knowledge:

-First study reporting and comparing the time associated with the scoring of respiratory events (both for the manual and semi-automatic approaches)

-Excluding preliminary estimations from our own group [32, 39], this is also the first study to report on time associated to manual and semi-automatic detection of leg movements and EEG arousals

-First study to provide results on the analysis of inter-scorer reliability under the context of semi-automatic scoring for the detection of leg movements, respiratory events, or EEG arousals.

-We have shown that even if the full-review semi-automatic sleep staging approach followed in this study might be suboptimal in terms of possible time gains (e.g. in comparison to partial quality-check guided as in Anderer et al. [10]), our data suggest that full-review of automatic sleep staging can still redound in cost savings regardless of what suggested in Svetnik et al. [20].

-First study to provide results on the analysis of inter-scorer reliability under the context of semi-automatic scoring for the detection of leg movements, respiratory events, and EEG arousals. That is, for event markings using kappa analyses. Available literature focusing on sleep staging only, or validation of full, i.e. not semi-automatic scoring.

-First study to report on scoring time and inter-rater reliability (both manual and semi-automatic, event markings and related indices) for detection of leg movements under recent WASM2016 standards.

-Our work contributes as well by adding to the existing literature on manual and semi-automatic scoring, taking into account that evolution of the scoring methods and reference clinical guidelines (i.e. new versions of AASM manual, WASM2016…) motivates periodic reassessment of the existing references, for which some of them might be outdated

They talk about “sleep stating” (only other instances they correctly refer to sleep staging), then they state in the abstract that they want to detect “EEG arousals”, but later refer to ”micro-arousals” or arousals alone. The use of different terms for the same or different types of arousals is confusing and needs to be defined.

The use of “sleep stating” instead of “sleep staging” in the abstract was a typo. It has now been corrected.

We have also carefully reviewed the manuscript and made changes to constraint as much as possible the variability of terms referring to the scoring of EEG arousals using a more consistent language.

The same is true for the “respiratory activity” (in the abstract), where they say respiratory events further down in the text, and even use the term “respiratory function”. If they just did done regular scoring of respiratory variables, they should call it as such and not as respiratory activity or respiratory function

Likewise, terminology regarding scoring of respiratory events has been reviewed.

The same is the identification of leg movements: it is okay to do leg movements, but they also should do PLM indices.

The use of consistent terminology for detection of leg movements has been reviewed as well.

Our study includes analysis of scoring time (Table 2), inter-rater agreement of individual leg movement event markings (Table 3) and derived indices (Table 4), the latter including PLM indices.

Furthermore, the authors conclude that the use of computer assisted scoring could help to reduce waiting lists of sleep laboratories: usually, the waiting lists of sleep laboratories are long not because technicians never finalize their scoring, but because of restrictions in polysomnography units and polysomnography beds

We absolutely agree with the reviewer that restrictions in polysomnographic units or beds might be a more important limitation factor than scoring time. Clinical setting is very complex and overall efficiency results from interconnection of several inter-dependent parts: availability of doctors, efficiency of appointments, electronic patient dossier, minimization of failed registrations, number and experience of technicians, communication, …. And it is also true that the specific bottle-neck of this responsibility chain can as well differ from center to center.

That said, in this study we focus on the part that regards the time dedicated to PSG review, and what we have found is evidence that this time can significantly be reduced (Table 2) with the help of semi-automatic scoring.

We have modified the discussion to lower down the focus on the mere reduction of the waiting lists (lines 372-374) and we have removed the reference from the concluding paragraph (550-552).

Once again we would like to emphasize that analysis of the scoring time was not the only focus of this study.

As a general remark, while it seems nice that they selected PSG for sleep staging, respiratory, LM etc., in today’s advanced background knowledge on automatic scoring one would at least expect that they define larger samples from well-defined patient and diagnostic groups.

We share as well the opinion of the reviewer in this aspect. Even though we initially aimed for a bigger sample, the lack of enough budget support, unfortunately, imposed a limitation on the availability of expert’s time preventing the possibility to add more recordings to the study. The issue is openly identified in the discussion as one important study limitation (lines 535-546).

In addition, we would like to refer to the implemented selection mechanism described in section “selection of PSGs” that aimed to minimize the chances of selection bias and to the relative high number of experts (12) rescoring each of the 20 selected PSGs.

The scoring algorithm should be made public, and not simply referred to dozens of own studies

We have added a link to a free-version of the Polyman software as well as GitHub repository with source code for the automatic EEG arousals’ scoring algorithm (lines 165-166). Unfortunately, the remaining algorithms are subject to license restrictions that do not allow for direct sharing of their source code.

We believe we should keep references to the corresponding published validation studies that describe these algorithms in detail as they contain the specifications that would enable their implementations, but in general, for the sake of completeness and scientific rigor. Additional discussion on this topic is provided in relation to a next related comment from the reviewer.

In addition, we have carefully scrutinized the overall number self-citing references in the manuscript and we were able to remove two of them.

There were several statements made, which are not backed up by evidence, for instance: “semi-automatic scoring, therefore, effectively results in valid …”

The corresponding paragraph has completely been rewritten to clarify the author’s point (lines 72-80).

Methods:
the authors used “PSG data for this study has been gathered by retrospective inspection…”, how can they make sure that this did not introduce a large bias?

The corresponding text has been rearranged referencing to the automatic selection procedure implemented with the aim to minimize the risk of selection bias.

It is also unclear what they mean with statements like “no patients was therefore subjected to any additional behavior in relation to this study, nor was prescribed any additional treatment outside of the regular clinical workflow”, this is superfluous, as in a retrospective study no patient will undergo any treatment or “additional behavior”

We agree, such “standard wording” is repetitive and has now been removed.

Line 84: in the present study a group of sleep technicians were prompted to review… did these sleep technicians have any type of certification background? Were they 12 technicians or other scorers?

All scorers were experienced sleep technicians with completed training certification. The text in the manuscript has been rewritten including this information.

Sleep scoring:
here, the authors present an exaggerated number of self-citations (eight), and even refer the reader “to check the corresponding references” (!). This is annoying, why the reader should bother to read the present manuscript, if he is referred to so many references to check?

As discussed in previous comments, while it is not the objective of the study to carry out performance validation of full automatic scoring, the underlying computer algorithms that were used for the first pass of the semi-automatic approach are still relevant. We are analyzing four different PSG scoring (sub)tasks in our study, and for each one there are several relevant publications that describe and validate these methods which have been developed throughout the course of several years. We aimed to restrict it to couple of the most relevant for each one, which inevitably resulted in the list of referenced publications. We have carefully attempted to restrict it even more. In this particular paragraph we were able to remove just one of them.

We believe referencing the work is the best solution to avoid unnecessary repetition that would only result in much longer manuscript (length of the manuscript was another concern of the reviewer). Moreover, any attempt to carry out a description of the underlying automatic methods would be vague and add very little in the context of this work. The focus of this manuscript should be kept in the declared objective of “investigating inter-scorer agreement and scoring time differences associated with visual and computer-assisted analysis of polysomnographic (PSG) recordings”. The relevant references are better sources of information for the reader interested in full automatic scoring.

What is the use of the present ms, only to report that scoring time can be reduced in a not further described small sample with respect to not further described scoring criteria and with a one of multiple algorithms?

Objectives of the study (not limiting to scoring time), sample size, and underlying algorithms using during the semi-automatic scoring pass, have been discussed already in earlier comments.

Scoring criteria are described in lines 115-117 and in the section “Rescoring task”. Some parts of these sections have also been rewritten aiming for better motivation and improved readability.

It is furthermore unclear why they selected fivexfour PSG from 2801 recordings.
So finally they selected 20 PSG recordings, on the basis of what?
This could be biased bias, or did they simply use the first best available?
Did they screen the 2801 or do they just want to express the magnitude of the database?

As stated on a previous related comment, the major limitation preventing the possibility to add more recordings to the study sample was the limited available resources, which is acknowledged in the discussion as one important study limitation (lines 535-546).

Several paragraphs of the methods section have been rewritten aiming for better motivation and justification of the selection procedures as well. In particular, we would like to refer to the automated mechanism described in section “selection of PSGs” that was implemented to minimize the chances of selection bias.

In summary, this is a very long manuscript, which adds very little new knowledge.
Some statements are not based on data, for instance, faster scoring means … the possibility to reduce the waiting list by the consequent increase in the scoring production: usually the limited availability of PSG is based on the limited number of PSG places, not on scoring time

We have modified the discussion to lower down the focus on the mere reduction of the waiting lists, in particular in the referenced sentence “faster scoring means…” (lines 372-374). We also have removed a similar comment from the concluding paragraph (550-552).

We hope that the remaining general concerns expressed in this paragraph had already been correctly addressed throughout the replies to earlier comments.

Also, the authors, while on the one side extensively self-citing, do not cite others: for instance: with only few examples addressing the case of other scoring tasks: this is a very general term, and not backed by any reference.

“…with only few examples addressing the case of other scoring tasks…” was meant to be an introductory sentence. Discussion followed on in the subsequent paragraphs discussing and comparing results with several other works from the literature.

We have rearranged the text using a structured and more focused discussion to avoid confusion, and added explicit references to the signaled paragraph. In addition, and we have increased the number of referenced works from the state-of-the-art.

As long as the authors do not make available their software, the results cannot be reproduced by others. One should keep in mind, that this is a single software, among hundreds of sleep scoring etc. softwares, and therefore the results cannot be generalized

As stated before, we have now included a link to a free-version of the Polyman software as well as GitHub repository with source code for the automatic EEG arousals’ scoring algorithm. Unfortunately, the remaining algorithms are subject to license restrictions that do not permit direct sharing of source code. Regardless, the referenced publications include detailed descriptions of the corresponding algorithms that would enable their implementation.

The discussion comments on the possible lack of result’s generalization due to possible dependencies of the underlying used software (lines 515-522).

REVIEWER 2

Reviewer #2: The authors analysed the possible benefits of semi-automatic PSG scoring of sleep staging, EEG arousals, respiratory events and limb movements, compared to manual visual approach. They provide quantitative metrics of performance regarding scoring time and inter-scorer agreement, showing the benefit deriving from semi-automatic scoring of the above mentioned PSG parameters and providing a quantification of the time saved in this way.
The manuscript is clearly written, data are extensively provided, results well and clearly presented, the discussion is complete and clear and limitations are acknowledged. I do not have any comment

We appreciate the positive feedback from the reviewer.

REVIEWER 3

Reviewer #3: This paper presents very important work on evaluating the effect of autoscoring in PSG sleep analysis, a field that is still ridden with quite some skepticism toward automation. In principle, this paper should be published, however, some major weaknesses still need to be addressed:

We appreciate the feedback from the reviewer. Changes that have been made to address the identified weaknesses, which are subsequently described.

- the authors do not seem to be fully aware of the state of the art, when judged by the references given in line 25. Systems that are actually used in the field are Morpheus (cited via Pittman et al.), Somnolyzer, Michelle and, most recently, Ensosleep/Ensodata (see also table 2 in Fiorillo et al.). Such systems deserve more attention when discussing the results of the authors' own algorithms. While it is true that time savings have not been published for any of those systems, Anderer et al., Neuropsychobiology 2010;62:250–264 did look into agreements between different sem-automatic scorings. The striking property of those results is the much higher agreement after correction than are achieved in this paper. This points to the fact that the biggest gains from autoscoring are obtained when scorers are NOT allowed to change results freely, but instead are trained to recognize where correction is necessary (e.g. because signal quality was bad). Applying the strategy that the authors have used in this paper instead seems to entail much more extensive changes than are necessary to achieve valid clinical results (pretty much like allowing one scorer to change the output of another expert scorer to their own liking) - see for instance, k=0.99 in Anderer et al., vs,. k=0.8 in table 3. Also Punjabi et al., Sleep 38(10), 2015, seems to point in a similar direction. Thus the results presented here might have an unfair bias against autoscoring, which should be mentioned in the discussion

We have rearranged citations in the referenced line of the introduction section, adding the missing ones suggested by the reviewer. The Michelle system was already referenced by the work of Malhotra et al. but included only in the discussion. We certainly knew of Somnozyler, and honestly I cannot explain how we could have skipped it in the first place. We apologize, it has now been fixed.

Both works of Anderer et al. and Punjabi et al. have been added as well to the discussion. In particular, a closer look to the work of Anderer et al. brought out that they had also examined scoring times in relation to sleep staging. These new data have now been added to the corresponding section of the new restructured discussion.

We have also commented on the (full-review, possibly suboptimal) semi-automatic strategy implemented in this work, in comparison to the “quality-check-guided” approach implemented in Somnolyzer (lines 339-403, 410-413, 437-440).

Regardless, we do not believe that the implemented strategy “might have an unfair bias against autoscoring”. Not necessarily at least, but rather the opposite, if compared to Somnolyzer’s approach, as their quality control check mechanism effectively results in most of the autoscored recordings (or periods of them) will no longer be subject to manual inspection for not being signaled by the established structured decision procedure (as it reads in the earlier Anderer et al., Neuropsychobiology 2005;51:115-133 paper). In other words, on the non-signaled periods/recordings it will be assumed that autoscoring was correct, and therefore will remain uninspected (and thus “just autoscored”). Rightly so, “only 4.3 and 3.5% of epochs were changed by the experts during quality control” easily achieving the high K = 0.99 reported in the 2010’s study. That also might explain the big (5-fold) scoring time gains reported in the same work. To be considered, K = 0.99 is well-beyond the reported levels of agreement of the same expert in two different instants of time (intra-expert variability) across the literature…although longer discussion would be required on this aspect, which probably is out of the scope of the review concerning our paper.

We do acknowledge limitations with regard to possible dependencies on the automatic scoring algorithms used in our work, which is also included in the discussion (lines 515-522).

- the other weakness is the rather low number of recordings (5) used in each comparison. While this is addressed in the discussion, it still is very unfortunate. For instance, this means that each ICC is calculated based on 5 samples only, which is rather questionable. Why weren't all 4 tasks (staging, arousals, apneic events, leg movements) performed on each recording, which would not only increase the sample sizes but also much more correspond to the reality in a sleep lab, where these tasks are never solved in isolation? If those additional scorings can no longer be added to the study, then what remains is a much larger weakness due to sample size than currently acknowledged in the paper

Each of the ICC values reported in Table 4 has been obtained on the basis of n = 60 data points, resulting from 12 different scorers and 5 recordings, for each of the examined tasks. In general, for each of the analyses, this number is indicated in the legend of each table and in the section “Analysis methods”

We have modified Table 4 adding 95% confidence intervals for ICC to provide additional support to the reported results. We have also simplified it removing all rows from the last columns that reported statistical analysis on the assumption of no a priori agreement (r0 = 0) as all these comparisons showed p < 0.0001. Comment on this result has been moved to the accompanying text.

We have also updated section “Selection of PSGs” adding some lines to provide a better explanation on why we have chosen to evaluate each task independently, and thus why independent per-task selection was preferred rather than recording-level selection (lines 168-175).

The acknowledged limitation on the constrained number of recordings analyzed per task has been highlighted as an “important” one.

- on the other hand, some of the high ICC values (compare, e.g., again against Punjabi et al.) for some of the endpoints suggests that due to the fact that all scorers came from the same lab leads to an overestimation of some agreements, which again blures the advantages of autoscoring somewhat. This, too, needs more acknowledgement.

We have examined the work of Punjabi et al. and the absolute values of ICC reported in comparison to those included in our study. The following comparison derives:

For AHI: ranging 0.84-0.96 (Table 2), ours 0.98-0.99.

For HI: ranging 0.56-0.94 (Table 2), ours 0.60-0.75.

For ArI: ranging 0.09-0.82 (Table 3), ours 0.66-0.76.

Hence, though absolute values of ICC for AHI in our study are above their ranges, the other two fit well in their intervals and show less dispersion.

Regardless, we would like to comment that, based in our experience, ICC seems to be very sensitive to specific study setup conditions. Therefore, rather than comparing absolute values of ICC among different studies, we find it more interesting, in general, to focus discussion on relative comparison within each study (e.g. are there significant manual vs. semi-automatic differences in one study, do these differences translate to the other study, etc). Hence, when possible, we followed this approach throughout our discussion, including findings from Punjabi’s et al. work.

Possible limitations due to all scorers belonging to the same lab have been acknowledged in the discussion (lines 502-506):

“…it is important to remark that absolute values of the various investigated performance scores are associated with one specific sleep lab. This study does not involve analysis of inter-scorer variability across multiple centers, and thus results might not generalize to other centers. In such scenario, the respective values of scoring agreement are expected to be lower in comparison due to the greater amount of variability involved...”

- Finally one must mention that the strategy to always let scorers perform visual scoring first and semi-automated scoring second, despite the 4 months of separation, introduces a bias. A randomized order would have been much preferred.

Reviewer has a valid point. We believe that 4 months is still a safe margin, and that chances of significant bias are really low in practice. Nevertheless the proposed design would have been preferable from a methodological point of view. We thank again the reviewer’s feedback and we take note for the next time. A comment has been added to the discussion in this regard (lines 512-514).

In summary, this is good and important work but hampered by considerable shortcomings that only partly can be overcome

Once again, we thank the reviewer’s feedback and hope that our comments and modifications have addressed the identified concerns. We believe they have had a positive contribution toward improving quality of the manuscript.
